# Estrogen and tamoxifen up-regulate FXYD3 on breast cancer cells: assessing the differential roles of ER α and ZEB1

**DOI:** 10.1186/s40064-015-1022-7

**Published:** 2015-06-06

**Authors:** Paul Herrmann, Susan M Aronica

**Affiliations:** Department of Biology, Canisius College, 2001 Main Street, Buffalo, NY 14208 USA

**Keywords:** FXYD3, Estrogen, Tamoxifen, ZEB1, Estrogen receptor alpha

## Abstract

Increased expression of the FXYD3 family of proteins has been associated with lung, colorectal, bladder and pancreatic cancers, and recent evidence suggests that elevated FXYD3 may promote tumor cell proliferation in breast cancer as well. However, factors involved in up-regulating the expression of FXYD3 in breast cancer have not been identified. We evaluated whether estrogen and the selective estrogen receptor modulator tamoxifen could regulate the expression of FXYD3 on breast cancer cells. Estrogen receptor (ER) α-positive MCF-7 and ER α-negative MDA-MB-231 human breast cancer cells used in our studies were treated with estrogen, tamoxifen or the combination of these agents. Relative expression of FXYD3 was assessed using fluorochrome-tagged antibodies and a fluorescence cytometer. We found that estrogen and tamoxifen, used alone or in combination, significantly increased FXYD3 on MCF-7 cells. FXYD3 levels did not increase compared to the control samples when ER α-negative 231 cells were treated with estrogen or tamoxifen, alone or in combination, indicating that ER α was required for the increased FXYD3 response. We showed that ER α associates with the transcription factor ZEB1 in MCF-7 cells, and that decreasing ZEB1 protein expression using siRNA disrupts the ability of estrogen, but not tamoxifen, to increase FXYD3 in MCF-7 cells. Our results indicate that there may be two mechanisms, both involving ER α and one requiring ZEB1, through which FXYD3 may be increased by estrogen and tamoxifen in breast cancer cells. Ongoing research endeavors are focusing on identifying cellular components through which estrogen and tamoxifen, alone or in combination, differentially regulate FXYD3 expression in human breast cancer cells.

## Background

Breast cancer is the second most common cancer among American women, affecting about 1 in 8 women in the United States during their lifetime (Siegel et al. 2014; American Cancer Society 2014). It was projected that over 240,000 new cases of breast cancer would be diagnosed in women in 2014. Breast cancer is the second leading cause of cancer related death in women (Siegel et al. 2014; American Cancer Society 2014). The disease state is defined as a malignant tumor that typically starts in the stroma tissue surrounding the mammary ducts and metastasizes to other areas of the body. With this, the cells lose control of communication, repair, and differentiation processes that are periodically activated in normal mammary cells. In particular, a loss of control in the expression and/or function of cell surface proteins can lead to issues with signaling and transport mechanisms that have a high incidence in cancer cells (Shou et al. 2004; Frasor 2003; Wu et al. 2011).

FXYD3 is a member of the FXYD protein family (Crambert et al. 2005). There are over 10 members of the FXYD family that have been identified and characterized in mammals, but FXYD3 is unique from the rest in that it possesses two transmembrane domains and maintains an uncleaved signal sequence within the protein (Yamamoto et al. 2009). FXYD3 has been shown to be located at the cell surface and in intracellular membrane compartments. FXYD3 isoforms modify the function of Na^+^/K^+^-ATPase, which is further supported by the discovery that parts of the FXYD3 signal overlaps with the α subunit of the Na^+^/K^+^-ATPase (Yamamoto et al. 2009). FXYD3 protein is expressed in a number of cells in normal, untransformed tissues, including the bladder, lung, stomach and skin, but has been reported to be highly overexpressed in many tumors such as breast, androgen-dependent prostate, pancreatic, and colon cancer (Yamamoto et al. 2009; Li et al. 2014). Overexpression of FXYD3 is also believed to be involved in the tumorigenesis and development of esophageal squamous cell carcinoma (Zhu et al. 2013). Although increased expression of FXYD3 is associated with various cancers, the relevant biological factors and their associated mechanisms of actions through which FXYD3 is increased in transforming and cancer cell systems have not been identified.

Several hormonal factors, including estrogen, have been implicated in promoting the development, growth or spread of breast cancer tumor cells (Speirs et al. 1999; Ali and Coombes 2002). Elevated levels of estrogen have been shown to promote uncontrolled cell growth through the disruption of cell signaling processes, and increased levels of this hormone are often associated with higher incidences of breast cancer (Kim et al. 2013). Numerous studies have demonstrated that the selective estrogen receptor modulator (SERM) tamoxifen reduces tumor recurrence and lowers the chance of contralateral breast cancer when used in combination with chemotherapy (Fisher and Costantino 1999; Jordan and O’Malley 2007). While tamoxifen is often viewed as antagonistic to estrogen action when used as a treatment for hormone-responsive breast cancer, the observation that tamoxifen can act as a partial estrogen agonist on gene expression and that women can also develop tamoxifen-resistant or tamoxifen-promoted tumors suggests that there may be one or more common pathways through which estrogen and tamoxifen potentially promote cancer development (Fisher and Costantino 1999; Jordan and O’Malley 2007). With this in mind, we examined whether exposure of cells to estrogen, alone or in combination with tamoxifen, could regulate FXYD3 expression in human breast cancer cells. We also set out to determine if regulation of FXYD3 expression by estrogen and/or tamoxifen was associated with the presence and action of the estrogen receptor (ER) α protein and the transcription factor ZEB1.

## Methods

### Cells and cell lines

Human mammary carcinoma cell lines MCF-7 and MDA-MB-231 (231 cells) were purchased from ATCC. MCF-7 cells were maintained on MEM. MDA-MB-231 cells were maintained on Leibowitz L-15 media. All media used for the maintenance of cancer cells were supplemented with 10% fetal bovine serum (FBS) and antibiotic/antimycotic solution (penicillin G, streptomycin, and amphotericin B). All cell lines were maintained in incubators at 37°C and 5% CO_2_ under hydrating conditions.

### Reagents

Cell culture reagents, including FBS, antibiotic/antimycotic solution, Trypsin-EDTA solution, MEM, Leibovitz L-15 media and Opti-MEM were GIBCO products purchased from Invitrogen/Life Technologies (Grand Island, NY, USA). Chemicals used in buffer preparations and the following specific reagents were purchased from Sigma Chemical Company (St. Louis, MO, USA): 17β-estradiol (estrogen; E_2_), tamoxifen (4-hydroxy-tamoxifen; Tam). Anti-human FXYD3, anti-human E-cadherin, anti-human ZEB1, and anti-human ER α primary and secondary antibodies and molecular weight markers for SDS-PAGE, and ZEB1 siRNa and siRNA transfection reagent were all purchased from Santa Cruz Biotechnology (Santa Cruz, CA, USA).

### Flow cytometry staining and analysis of FXYD3 and E-cadherin surface expression

For experiments evaluating FXYD3 and E-cadherin surface expression, MCF-7 or 231 cells were treated with control vehicle [phosphate buffered saline, (PBS), or PBS containing 0.1% ethanol] or with the following reagents, alone or in combination, for 48 h: 1 nM 17-beta-estradiol (E_2_), 10 nM Tamoxifen (Tam). Treated cells were removed from flasks by trypsin-EDTA, pelleted by centrifugation (600×*g*, 5 min), and resuspended in residual media. Blocking antibody (5 μl of unlabeled, normal hamster IgG) was added to each tube and put on ice for 15 min. Tubes were gently vortexed every 5 min. Following the blocking step, each tube containing the entire treated cell population was split evenly by volume into three tubes, referred to as groups 1–3. Group 1 cells received the hamster blocking antibody only, group 2 cells received isotype antibody, and group 3 received specific anti-human FXYD3 or anti-human E-cadherin primary antibody. The specific primary antibody (FXYD3 or E-cadherin) was added to group 3 tubes and set on ice for 15 min, with vortexing every 5 min. Phycoerythrin (PE)-labeled secondary antibody was added to both tubes 2 and 3 and the tubes were set on ice for 15 min. Cold PBS (3 ml) was added to all tubes and cells were pelleted at 600×*g* for 5 min. PBS was decanted from all tubes and 500 µl of PBS was added to the cell pellet in each tube. Cells were resuspended and the samples were transferred to microcentrifuge tubes and analyzed for FXYD3 or E-cadherin expression using a Guava PC flow cytometer and Guava Express software.

### Immunoprecipitation

MCF-7 cells were treated with various reagents for 24 or 48 h, and then harvested. Cell pellets were resuspended in NP-40 lysis solution (1% NP-40 in PBS) and vortexed, to form cell lysates. Lysates were maintained on ice. Protein content of cell lysate samples were determined through the use of a Pierce BCA Protein Assay Kit with a bovine serum albumin (BSA) standard curve (Pierce Chemical Co., Rockford, IL, USA). For immunoprecipitation of ZEB1 proteins, aliquots of cell lysates were placed into microcentrifuge tubes on an equivalent total protein basis in order to equalize the amount of protein in the samples that were to be immunoprecipitated. Adjusted volumes (so that each tube contains the same amount of total protein) were added to microcentrifuge tubes from each lysate and 300 μl of 20 mM tris-buffered saline (TBS, pH 7.5) was added to each tube. Anti-human ZEB1 primary antibody (10 μl; 2 μg) was added to each sample and allowed to mix on a rotator, end-over-end, in a 4°C cold room for 1 h. Protein G-Sepharose beads were resuspended in TBS and 100 μl of bead slurry was added to each tube and tubes allowed to mix for an additional 1 h on a rotator in the cold room. The samples were then centrifuged (600×*g*, 5 min, 4°C) to pellet the beads and the supernatant was discarded. The bead pellet in each tube was washed with 100 μl of 1% NP-40 lysis buffer and 200 μl of 20 mM TBS buffer and mixed on a rotator in the cold room for 10 min. These samples were centrifuged and the supernatant was discarded. Two more wash steps were carried out using 300 μl of 20 mM TBS and the bead pellets were kept each time. After the final wash, 20 μl of loading buffer containing β-mercaptoethanol (BME) was added to each sample. Just prior to loading SDS-PAGE gels, samples were centrifuged briefly at high speed to pellet the Sepharose beads.

### Protein analysis by SDS-PAGE and western immunoblotting for estrogen receptor

Following immunoprecipitation of ZEB1 proteins and removal of the protein-G beads, immunoprecipitated samples were boiled for 3 min and then loaded onto 10% polyacrylamide gels. Proteins were separated according to molecular weight by SDS-PAGE under reducing conditions and transferred to PVDF membranes by iBlot turboblotter. Membranes were subsequently immunoblotted with rabbit anti-human ER α primary antibodies at a concentration of 1:200 in a 5% blocking solution (5% carnation non-fat dry milk in 20 mM TBS, pH 7.5). Secondary antibodies (goat anti-rabbit horseradish peroxidase linked) were used at a concentration of 1:2000 in the same 5% blocking solution. Membranes were washed three times in TBST (TBS containing 0.1% tween 20). Washed membranes were then exposed to BIO-RAD Clarity luminol-containing reagents and imaging was carried out on a digital Versadoc imager. The digital images were then examined for banding patterns for proteins and corresponding molecular weight markers using Multi-analyst software.

### Transfection of breast cancer cells with ZEB1 siRNA

We were interested in assessing the potential role of the ZEB1 protein in mediating the regulation of FXYD3 expression by estrogen and tamoxifen. Towards that goal, we transfected cells with siRNA directed against human ZEB1 in order to decrease ZEB1 expression. MCF-7 cells were grown in 6-well plates until 50% confluency. Maintenance media was replaced with antibiotic free Opti-MEM for 24 h prior to transfection. Transfection of the cells was carried out using Santa Cruz Transfection Reagents. Working under a sterile tissue culture hood, Opti-MEM was exchanged for 1 ml of serum-free, antibiotic free Opti-MEM for each well and plates were placed in the incubator while siRNA-lipid complexes were prepared. For each well to be transfected, two microcentrifuge tubes, one for diluting the siRNA (Solution A) and one for diluting the lipid transfection reagent (Solution B) were prepared. Solution A tubes contained 100 μl of Opti-MEM and 6 μl of the ZEB1 siRNA. Solution B tubes contained 100 μl of Opti-MEM and 6 μl of transfection reagent. For each pair of tubes, the A and B tubes were combined by transferring Solution A slowly drop-wise into Solution B. The combined contents now in tube B were gently mixed up and down with a micropipette to insure a uniform reaction of the reagents. The combined solution was incubated at room temperature (RT) for 25 min.

After the incubation period, 800 μl of Opti-MEM was added to the combined A and B tube and gently mixed with a pipette. At this point the plates were removed from the incubator and the antibiotic free media was removed from the transfection wells. The entire 1 ml combined volume containing the transfection siRNA-lipid complexes were added to the respective wells in a drop-wise fashion to overlay the cells. The cells were incubated overnight for 24 h and then the media was changed to 1 ml of media containing serum and antibiotics. Transfected cells were treated with PBS/ethanol vehicle, estrogen, tamoxifen, or the combination of estrogen and tamoxifen for 24 h. Flow cytometry was used to analyze FXYD3 levels of treated transfected cells, as described. For comparison controls for various experiments, some wells of cells were left untransfected, some were mock transfected where they were exposed to all transfection reagents but not siRNA, and some were transfected with non-targeting siRNA.

### Statistical analyses

FXYD3 expression data were statistically analyzed by ANOVA using Microsoft Excel Software. Significant difference between pairs of treatment groups was determined by paired Student’s t tests. Differences between treatment groups at a level of p < 0.05 was considered to be statistically significant.

## Results

### Estrogen and tamoxifen exposure increases cell surface expression of FXYD3

**Figure 1:**
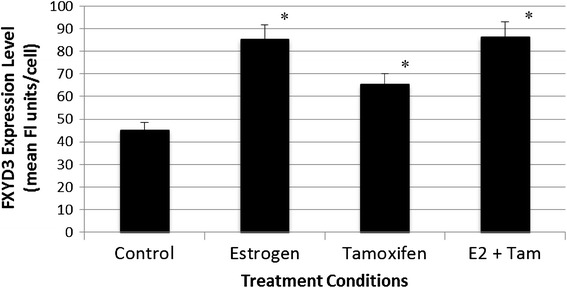
FXYD3 levels in MCF-7 cells. MCF-7 cells were treated for 48 h with PBS/0.1% ethanol control vehicle, 1 nM estrogen, 10 nM tamoxifen or with estrogen in combination with tamoxifen, and expression of FXYD3 was determined by flow cytometry using anti-FXYD3 antibodies and a Guava PC flow cytometer, as described in the “Methods”. Each *bar* represents the mean ± SD of FXYD3 fluorescence per cell for six samples obtained in separate experiments. *Asterisk* significant difference (p < 0.05) from PBS/ethanol-treated control cells.

**Figure 2:**
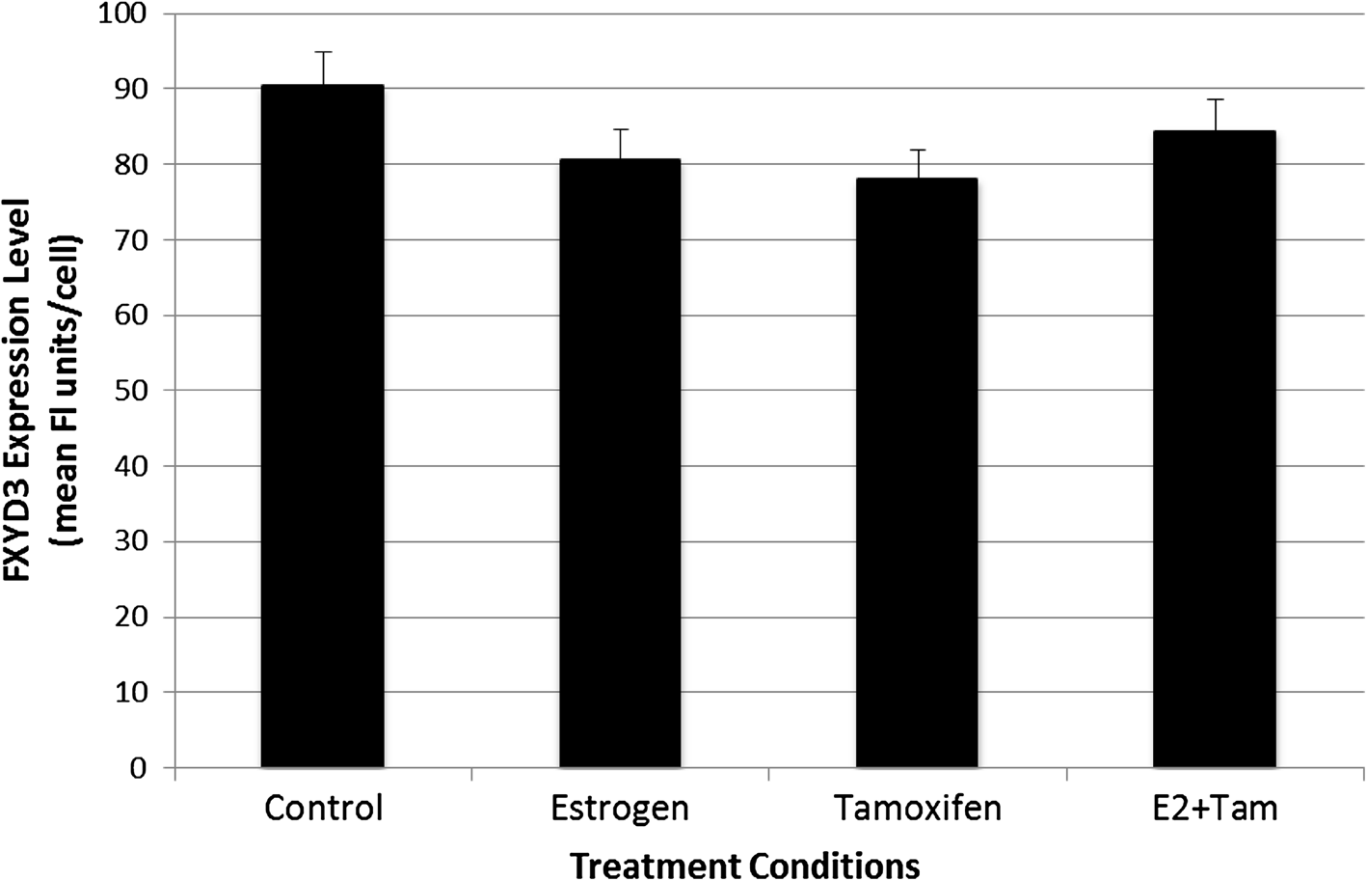
FXYD3 levels in MDA-MB-231 cells. MDA-MB-231 cells were treated for 48 h with PBS/0.1% ethanol control vehicle, 1 nM estrogen, 10 nM tamoxifen or with estrogen in combination with tamoxifen, and expression of FXYD3 was determined by flow cytometry using anti-FXYD3 antibodies and a Guava PC flow cytometer. Each *bar* represents the mean ± SD of FXYD3 fluorescence per cell for six samples obtained in separate experiments.

We set out to evaluate whether estrogen and tamoxifen could affect the expression of FXYD3 or E-cadherin on the surface of MCF-7 and 231 breast cancer cells. MCF-7 or 231 cells maintained in culture were treated with PBS/0.1% ethanol vehicle alone (PBS), or with 1 nM estrogen or 10 nM tamoxifen alone, or with these reagents in combination, for 48 h. Cells were harvested and FXYD3 expression was determined by flow cytometry, as described in the “Methods”. All treatment agents significantly increased cell surface expression of FXYD3 in MCF-7 cells when compared to PBS/ethanol control (Figure 1). In contrast to results obtained with ER-positive MCF-7 cells, exposure of ER-negative 231 cells to estrogen, tamoxifen or the combined treatment failed to evoke increases in FXYD3 expression, resulting in FXYD3 levels at or below those of control cells (Figure 2). Treated MCF-7 cells were also evaluated for altered expression of E-cadherin. In contrast to results obtained for FXYD3 expression, exposure of MCF-7 cells to 1 nM estrogen, 10 nM tamoxifen or the combination of agents for 48 h failed to evoke changes in cell surface expression of E-cadherin when compared to control, vehicle-treated cells (data not shown).

### ZEB1 associates with ER in MCF-7 cells

**Figure 3:**
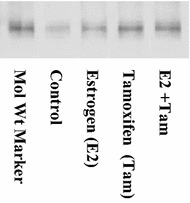
ZEB1 associates with estrogen receptor in MCF-7 cells. MCF-7 cells were treated with PBS/0.1% ethanol control vehicle, 1 nM estrogen, 10 nM tamoxifen or with estrogen in combination with tamoxifen for 24 h. ZEB1 was immunoprecipitated from cell lysates using anti-ZEB1 antibodies, as described in “Methods”. Immunoprecipitated proteins were separated by SDS-PAGE and transferred to PVDF membranes. Presence of ER α in immunoprecipitated samples was determined by Western immunoblotting using anti-ER α antibodies, as described in “Methods”. Imaging of membranes was performed with a Versadoc imager and Multi-analyst software.

We were interested in determining whether the transcription factor ZEB1 associated with ER α in the presence or absence of estrogen or tamoxifen in MCF-7 cells. MCF-7 cells maintained in culture were treated with PBS/ethanol vehicle (control), 1 nM estrogen, 10 nM tamoxifen or with estrogen in combination with tamoxifen for 48 h. ZEB1 proteins were immunoprecipitated from cell lysates in non-reducing conditions using anti-human ZEB1 antibodies, as described in the “Methods”. Proteins from immunoprecipitated samples were separated according to molecular weight by SDS-PAGE and transferred to PVDF membranes. Membranes were then probed with anti-ER α primary and HRP-labeled secondary antibodies and subjected to image analysis for banding patterns. Our results indicate that the ZEB1 protein associates with ER in control treated cells, as evidenced by the presence of ER α in the ZEB1-immunoprecipitated control cell lysate (Figure 3, lane 2). This association between ZEB1 and ER was maintained, and in some instances appeared to increase, as evidenced by the darker ER bands, for cells treated with estrogen, tamoxifen or the combination of treatments (Figure 3).

### ZEB1 protein is required for up-regulation of FXYD3 expression by estrogen

**Figure 4:**
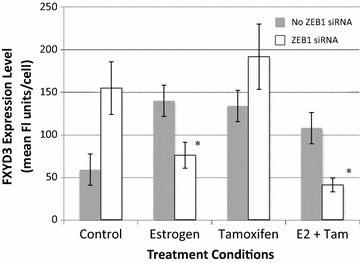
Effect of decreased ZEB1 expression on FXYD3 expression. Decreasing ZEB1 expression was achieved by transfecting MCF-7 cells with siRNA targeting human ZEB1. Following the transfection procedure, cells were treated with control PBS/ethanol vehicle, or with estrogen, tamoxifen or with estrogen in combination with tamoxifen for 24 h. Expression of FXYD3 on cells was determined by flow cytometry using a Guava PC instrument and Guava Express software. Each *bar* represents the mean ± SD of FXYD3 fluorescence per cell for six to eight samples obtained in separate experiments. FXYD3 expression data from untransfected or mock transfected (non-targeting siRNA) cells were pooled together. *Asterisk* significant difference from ZEB1 siRNA transfected, control-treated cells.

With the observations that estrogen exposure increased expression of FXYD3 in MCF-7 breast cancer cells, and that exposure of these cells to estrogen increased the association of ER with the ZEB1 protein, we set out to determine whether declines in ZEB1 levels would alter the ability of estrogen or tamoxifen to regulate FXYD3 expression. MCF-7 cells maintained in culture were either left untransfected, were mock transfected with non-targeting reagents, or were transfected with human ZEB1 siRNA in order to decrease ZEB1 levels, as described in the “Methods”. Following the transfection procedure and treatment of cells with PBS/ethanol control vehicle, 1 nM estrogen, 10 nM tamoxifen or the combined treatment of estrogen and tamoxifen for 36 h, cells were harvested and FXYD3 expression was determined by flow cytometry. As with our initial experimental results, exposure of untransfected or mock transfected MCF-7 cells (data combined) to estrogen, tamoxifen or the combination of estrogen and tamoxifen increased expression of FXYD3 when compared to the PBS/ethanol control (Figure 4). Of particular interest, however, is that upon transfection with ZEB1 siRNA, expression levels of FXYD3 in PBS/ethanol vehicle-treated control cells increased substantially (Figure 4). Moreover, exposure of these ZEB1 siRNA transfected cells to estrogen or estrogen in combination with tamoxifen promoted a substantial decline, rather than increase, in FXYD3 expression (Figure 4). In contrast to the estrogen exposed cells, exposure of ZEB1 siRNA transfected cells to tamoxifen alone did not result in substantial increases or decreases in FXYD3, but with cells maintaining levels of FXYD3 at or slightly above those elevated levels detected for vehicle-treated ZEB1 siRNA transfected cells (Figure 4).

## Discussion

The FXYD genes encode for proteins that function to regulate the transport functions of N^a+^/K^+^ ATPases in a variety of normal cell systems (Crambert et al. 2005; Yamamoto et al. 2009). One member of this family, FXYD3, not only serves to regulate ATPase function in normal physiological systems, but several studies have shown that overexpression of this member of the FXYD family is associated with a number of cancers, and has been implicated in direct action in the formation of at least one type of cancer (Yamamoto et al. 2009; Li et al. 2014; Zhu et al. 2013; Grzmil et al. 2004). While increased expression of FXYD3 has been associated with breast cancer, the factors that promote up-regulation of this protein in mammary cells have not been identified. We report in the results of our current study that both estrogen and the SERM tamoxifen can significantly increase the expression of FXYD3 in ER α-positive, MCF-7 human breast cancer cells. Moreover, the highest levels of FXYD3 were detected in cells exposed to both estrogen and tamoxifen. To our knowledge, this is the first report showing that FXYD3 can be increased by estrogen and tamoxifen in breast cancer cells. Our observation that up-regulation of FXYD3 by either factor was not observed in ER α-negative 231 cells suggests that the effect of estrogen and tamoxifen on the expression of FXYD3 requires the presence of the α isoform of the ER protein.

Our observation that estrogen and tamoxifen could serve to increase FXYD3 expression in breast cancer cells prompted us to examine potential mechanisms through which this increased gene expression might be occurring. Recent reports had shown that the transcriptional repressor ZEB1 mediated the down-regulation of FXYD3 in human mammary cells that had been exposed to transforming growth factor beta (TGF beta) (Yamamoto et al. 2011). The authors went on to show that the silencing of ZEB1 expression prompted an increase in FXYD3 in cells that had been suppressed by TGF beta (Yamamoto et al. 2011). Since estrogen has been known to regulate gene expression through alteration of gene transcription (Kim et al. 2013; Jordan and O’Malley 2007), we set out to determine whether the increases we observed in FXYD3 for cells exposed to estrogen or tamoxifen could be associated with the action of ZEB1. We initially sought to see if the ER protein and ZEB1 proteins actually interacted in our MCF-7 cells. Results of immunoprecipitation experiments showed that ER α and ZEB1 associated with one another in PBS-treated cells, and that this interaction was maintained or increased in the presence of estrogen, tamoxifen or the combination of these agents. We then sought to determine if there was a central role for ZEB1 in the up-regulation of FXYD3 by decreasing the ZEB1 protein and seeing if the base levels and hormone-influenced levels of FXYD3 were changed as a result. Using siRNA directed against human ZEB1, we lowered the amount of ZEB1 in transfected MCF-7 cells and then assessed cells for basal levels of FXYD3 and evaluated whether suppression of ZEB1 altered cellular responses to estrogen and tamoxifen. We found that the mere suppression of ZEB1 protein levels in MCF-7 cells served to promote an increase in FXYD3 protein levels. Treatment of these cells with estrogen or estrogen in combination with tamoxifen failed to increase FXYD3 and, in fact, tended to evoke decreases in FXYD3 to levels below those of the control cell population. In contrast, treatment of cells transfected with ZEB1 siRNA with tamoxifen alone, in the absence of additional estrogen, maintained elevated FXYD3 levels consistent with or slightly above those of the control PBS treated transfected cells.

Our observations provide the first evidence of a mechanism through which estrogen can serve to up-regulate FXYD3 in human breast cancer cells. The ability of estrogen to increase FXYD3 expression appears to require both the presence of ER α and ZEB1 in the MCF-7 cell system. It is of interest that the suppression of ZEB1 proteins in MCF-7 cells suppresses the ability of estrogen to increase FXYD3, suggesting that the presence of the ZEB1 protein is integral to the action of ER α in enhancing expression of FXYD3. Estrogen, working through ER α, has been shown to up-regulate the expression of ZEB1 in MCF-7 cells, suggesting that increased ZEB1 levels are related to the pro-cancer phenotype promoted by estrogen action within these cells (Guo et al. 2012). Whether the action of estrogen to increase FXYD3 expression, mediated by ER α, involves direct activation or inactivation of ZEB1, and/or involvement of other transcriptional regulators has yet to be determined. Other groups have shown that activation of ZEB1 results in the active repression of the expression of other genes, including those that are involved in cell polarity differentiation of epithelial cells and those that are involved in EMT in cancer development (Spaderna et al. 2008; Aigner et al. 2007). In addition, a separate report indicates that ER α and ZEB1 work in opposition to one another in controlling the expression of microRNAs in breast cancer cells (Simonini et al. 2010). Our observation that decreasing ZEB1 results in increased levels of FXYD3 expressed in control, vehicle-treated cells could be indicative of a block to, or reversing of, repression of gene expression by ZEB1, consistent with results of previously published studies (Yamamoto et al. 2011). Of particular interest in our own findings, however, is that the decline in ZEB1 appears either to prevent up-regulation of FXYD3 by estrogen and/or alters the action of the estrogen–ER α interaction to act as a suppressor of FXYD3 expression, while simultaneously permitting tamoxifen to promote or maintain elevated FXYD3 protein levels in this cell system. This indicates that the SERM tamoxifen acts more like an estrogen agonist rather than antagonist on FXYD3 expression in either the presence or absence of ZEB1. This is in contrast to results of another study that suggests that the presence of ZEB1, and not the absence of ZEB1, is associated with tamoxifen resistance in breast cancer cells (Guo et al. 2012). There is some evidence in uterine cell systems that ZEB1 may be positioned on chromatin in target genes in at least three conformations (repressive, poised, active), and perhaps this potential for at least three conformations of ZEB1 might be relevant to the differences we see in how regulation of ZEB1 protein level affects the ability of estrogen and tamoxifen to regulate FXYD3 (Spoelstra et al. 2006). In fact, our observation that tamoxifen can promote or maintain elevated levels of FXYD3 in the absence or diminished levels of ZEB1, contrary to the results obtained with estrogen exposure, might give some insight into a potential avenue through which tamoxifen might contribute to cancer progression, rather than suppression, in ER α-positive breast cancers. Ongoing research efforts are focusing on determining what specific transcriptional factors are involved in the regulation of FXYD3 expression in breast cancer cells by estrogen, tamoxifen, and the combination of these agents.

## Conclusions

Our study provides the first primary evidence of estrogen and the SERM tamoxifen serving as up-regulators of FXYD3 on human breast cancer cells. Our study also suggests that the mechanisms through which estrogen and tamoxifen upregulate FXYD3 differ from one another, with both agents appearing to require the presence of ER α, but with estrogen action also requiring the presence of ZEB1. Our future studies will focus on the mechanisms through which these factors influence expression of FXYD3 on human breast cancer cells, and analysis of how such changes in FXYD3 evoked by estrogen or tamoxifen affect proliferative activity of these cells.
